# FACILITATORS OF AND BARRIERS TO ADULT DAY SERVICES PROVISION: PROVIDERS’ PERSPECTIVES

**DOI:** 10.1093/geroni/igae098.3842

**Published:** 2024-12-31

**Authors:** Kingsley Udeh, Molly Noble, Heather Menne, Randi Hamill, Sara McLaughlin

**Affiliations:** MIAMI UNIVERSITY OHIO, oxford, Ohio, United States; Miami University, Oxford, Ohio, United States; Miami University, Miami University, Ohio, United States; LeadingAge Ohio, Columbus, Ohio, United States; Miami university, Oxford, Ohio, United States

## Abstract

Adult day services (ADS) can help people living with various chronic conditions remain in their community, and may improve well-being among participants and their family caregivers. Despite the potential benefits, knowledge of the ADS landscape is underdeveloped, especially as it pertains to the experiences of ADS providers since the COVID-19 pandemic. This study aimed to understand the facilitators and barriers experienced by ADS providers. We conducted two focus groups with seventeen ADS providers in Ohio. Thematic analysis was used to identify themes in the data. Several facilitators for providing ADS were identified, including dedicated, consistent, and committed staff; partnerships with local organizations; sufficient reimbursement; and diverse funding sources. Barriers included: staffing issues (e.g., recruitment and retention); referral and recruitment of ADS participants; limited knowledge, awareness, and misconceptions about ADS among families and case managers; transportation services (e.g., inadequate and safe escort of participants to and from ADS centers); and insufficient or delayed reimbursement. Providers identified the following solutions with the potential to mitigate perceived barriers: educational initiatives to foster understanding of ADS among key stakeholders (e.g., regulators, case managers, community members) and additional funding and media publicity support from local, state, and federal authorities. As one of the only studies that seeks to understand the landscape of ADS in Ohio, this research is uniquely situated to provide crucial insights for tailoring interventions that consider ADS providers’ unique challenges. It also informs future research focusing on improving the quality of ADS.

